# How are adults with intellectual and/or developmental disabilities represented, included and engaged in cancer research: A scoping review protocol

**DOI:** 10.1371/journal.pone.0346010

**Published:** 2026-04-15

**Authors:** Rebecca L. W. Hansford, Sandra Halliday, Nicole Bobbette, Laura Mullins, Christina Godfrey, Anna Przednowek, Preeyaporn Srasuebkul, Martin McMahon, Alyson L. Mahar

**Affiliations:** 1 School of Nursing, Queen’s University, Kingston, Ontario, Canada; 2 Division of Cancer Care and Epidemiology, Queen’s University, Kingston, Ontario, Canada; 3 Queen’s University Library, Queen’s University, Kingston, Ontario, Canada; 4 School of Rehabilitation Sciences, Queen’s University, Kingston, Ontario, Canada; 5 Department of Applied Disability Studies, Brock University, St Catharines, Ontario, Canada; 6 JBI Queen’s Collaboration for Health Care Quality, (Queen’s University, Kingston, Canada), University of Adelaide, Australia; 7 School of Social Work, Nipissing University, North Bay, Ontario, Canada; 8 School of Medicine and Health; University of New South Wales, Sydney, Australia; 9 School of Nursing and Midwifery; Trinity College Dublin, Dublin, Ireland; Harvard University, UNITED STATES OF AMERICA

## Abstract

Cancer research has shifted to highlight and empower the voices and experiences of patients in informing and guiding research. Adults with intellectual and/or developmental disabilities experience worse cancer-related outcomes compared to those without disabilities, including lower screening rates, more advanced stages at diagnosis, and lower survival likelihood. However, limited research has been conducted on how they are involved in cancer research. To improve how cancer-related care is provided to adults with intellectual and/or developmental disabilities, it is imperative that they are included in research that affects them. The objective of this scoping review is to explore and synthesize how adults with intellectual and/or developmental disabilities are represented, involved, and engaged in cancer research. This study will follow the JBI methodology for scoping reviews. Embase, Medline, PsycINFO and CINAHL will be searched to identify published studies. Based on timing of the introduction of the United Nations on the Rights of Persons with Disabilities, searches will be conducted from 2006 onwards. There will be no language restrictions. Eligible studies will include primary cancer-related research that focuses on adults with intellectual and/or developmental disabilities and/or those involved in their care, including qualitative, quantitative, and mixed methods research. Two independent reviewers will screen titles, abstracts, conduct full text reviews, and extract information on how participants were recruited in research, and/or identified in administrative quantitative studies, and how they were involved. Information will also be extracted on how adults with intellectual and/or developmental disabilities were involved in the study based on the International Association for Public Participation spectrum (inform, consult, involve, collaborate, and empower). Our findings will help us better understand how adults with intellectual and/or developmental disabilities are engaged in cancer research and identify potential next steps for enhancing accessibility and inclusion in cancer research.

## Introduction

Adults with intellectual and/or developmental disabilities often experience worse cancer-related care and outcomes than those without disabilities [1–6]. People with intellectual and/or developmental disabilities are more likely to be diagnosed with incurable cancer compared to those without intellectual and/or developmental disabilities and are significantly more likely to die of their cancer [1,3,7,8]. These disparities are likely driven, in part, by lower cancer screening uptake and a lack of accessible, inclusive cancer prevention strategies [3–5,9–12]. In addition, when diagnosed with the same stage of cancer at diagnosis, adults with intellectual and/or developmental disabilities receive different cancer treatments than those without intellectual and/or developmental disabilities [7,13].

Adopted in 2006, the United Nations Convention on the Rights of Persons with Disabilities (UNCRPD) emphasizes the need to promote, protect, and ensure equal and full human rights and freedoms for people with disabilities, while also promoting respect for their dignity [14]. Article 25 of the UNCRPD highlights how persons with disabilities have the right to the highest standard of health without discrimination based on their disability [14]. However, barriers to cancer care and consequently poorer outcomes relative to cancer patients without disabilities have persisted among persons with intellectual and/or developmental disabilities. There is a need to better understand how to effectively support adults with intellectual and/or developmental disabilities receiving cancer-related care as well as others involved in their care (e.g., family members, paid/unpaid caregivers, and healthcare professionals), including a focus on the provision of accessible and inclusive person-centred care.

Person-centred care prioritizes knowing patients as persons, and ensuring that they are guiding care decisions [15]. In recent years, there has been a shift from the Golden Rule (“do unto others as you would have them do unto you”) to the Platinum Rule (“doing unto patients as they would want done unto themselves”) [15,16]. We cannot meaningfully improve the accessibility of cancer care or fully appreciate the extent of these barriers if those experiencing barriers are not included in research. While cancer advocacy has improved over time for cancer patients without disabilities and family members [17–19], we do not yet know the extent to which adults with intellectual and/or developmental disabilities or others involved in their care (e.g., paid/unpaid caregivers, etc.) are included in such research as no reviews have focused on the representation of this patient population in cancer research [7,20–34]. This scoping review will explore if and how adults with intellectual and/or developmental disabilities, and those involved in their care, including family members, paid/unpaid caregivers, and healthcare professionals, are represented, included, and engaged in cancer research.

## Scoping review questions

How are adults with intellectual and/or developmental disabilities represented, involved, and engaged in cancer research?

### Representation

1. How are adults with intellectual and/or developmental disabilities identified in administrative data sources (e.g., medical only, social services, education) used in population-based cancer research. Which diagnoses are included in these algorithms? What detail are available on their underlying health condition interacting with systems, people and environment to cause disability? What information is considered on the supports they receive, in which settings.

### Involvement

2. How are adults with intellectual and/or developmental disabilities recruited in non-administrative data studies? Are they themselves specifically recruited and consented, or are those involved in their care/caregivers recruited? Are caregivers (paid and unpaid, including family members and support workers) recruited as proxy respondents for adults with intellectual and/or developmental disabilities and/or for reporting their experiences of caring for an individual with intellectual and/or developmental disabilities and cancer? How might recruitment strategies be biased or rely on gatekeeping?
3. How are healthcare professionals recruited to report on their experiences of providing cancer care to adults with intellectual and/or developmental disabilities and cancer?

### Engagement

4. How are adults with intellectual and/or developmental disabilities and/or their carers (family members, paid/unpaid caregivers, and healthcare professionals) engaged in the research (e.g., as research participants, as proxies, or as co-researchers/advisors)?
5. How are adults with intellectual and/or developmental disabilities and/or their carers (family members, paid/unpaid caregivers, and healthcare professionals) engaged in cancer research based on the International Public Participation spectrum [35] (are co-researchers/advisors informed, consulted, involved, collaborated with, or empowered)?

## Materials and methods

This scoping review will be conducted in accordance with JBI methodology for scoping reviews [36], based on the Preferred Reporting Items for Systematic Reviews and Meta-analyses Extension for Scoping Reviews (PRISMA-ScR) [37]. This protocol has been registered on Open Science Framework (https://osf.io/gmyp6/metadata/osf).

### Patient and public involvement

For the development of this protocol, we consulted with two paid advisors: 1) an adult with intellectual and/or developmental disabilities who has undergone participated in cancer screening, 2) a parent of an adult child with intellectual and/or developmental disabilities with ovarian cancer. The advisors provided initial feedback on the aims of the scoping review. We will meet regularly with both advisors throughout the duration of the scoping review, including while completing the narrative summary and when developing strategies for accessible and inclusive knowledge sharing recommendations from the findings. Co-authorship or formal acknowledgement in the full review publication will be offered to reflect their contributions to the interpretation of the data.

## Eligibility criteria

### Participants

This review will include studies involving adults aged 18 years and older with intellectual and/or developmental disabilities receiving preventive and cancer-related care (e.g., see relevant International Classification of Disease 9 and 10 codes in S1 Table). Intellectual and/or developmental disabilities refer to conditions that originate during the developmental period, affecting intellectual functioning, social skills, and adaptive functioning [38]. Disability arises based on the interaction between persons with conditions, and societal barriers, including physical and environmental barriers as well as systemic systems of oppression, such as negative attitudes and bias [39]. We will also include studies involving family members, caregivers, support workers, or healthcare professionals providing support to adults with intellectual and/or developmental disabilities receiving cancer-related care. Eligibility will focus on research conducted at various points along the cancer control continuum, including cancer awareness/literacy, screening, incidence, stage at diagnosis, treatment, survival, survivorship, psycho-social oncology, supportive care, and end-of-life care. For studies with samples of adults with a mixed range of disabilities (i.e., physical and intellectual), we will only include studies that report disagregated data for those with intellectual and/or developmental disabilities.

### Concept

This review will consider studies that explore cancer outcomes and experiences of adults with intellectual and/or developmental disabilities. The concept will also include the experiences of family members, caregivers, support workers, or healthcare professionals providing support to adults with intellectual and/or developmental disabilities through their cancer experience.

### Context

The context for this review is the direct or indirect experience of care and outcomes related to cancer including awareness/literacy, screening, incidence, stage at diagnosis, treatment, survival, survivorship, psycho-social oncology, supportive care, and end-of-life care among adults living with intellectual and/or developmental disabilities.

### Type of evidence sources

The review will consider quantitative, qualitative, and mixed methods study designs. Quantitative studies will include clinical trials, quasi-experimental studies, and observational research. Qualitative studies will consist of various study traditions, including participatory action research, ethnographies, case studies, phenomenology, and grounded theory. Mixed methods studies will be included when components of the qualitative or quantitative findings can be extracted separately.

### Exclusion criteria

We will exclude studies that do not provide specific outcomes or experiences for adults with intellectual or developmental disabilities (e.g., disability generally), focus on pediatric cancers (age < 18 years), as well as reviews, editorials, case reports, and non-peer reviewed resources (e.g., dissertations, conference abstracts).

### Search strategy

An initial search was conducted by an academic health sciences librarian (SH) in Medline, Embase, PsycINFO and CINAHL using relevant subject headings and text words that corresponded to the main topics in the research question. Two limits were applied to the search strategy that matched to the inclusion/exclusion criteria for the research project, namely adult population and articles published from 2006 to 2025. The librarian checked that five key studies were identified with the search terms. The search is provided in Supplementary materials (S2–S5 Tables). This search was peer-reviewed by a second librarian based on the Peer Review of Electronic Search Strategies (PRESS) guidelines [40]. Two reviewers will also examine the reference lists of included articles for any missed relevant citations. These articles will be screened and reviewed in full as needed. Only articles from 2006 and onwards will be included based on the adoption of the UNCRPD [14]. There will be no language restrictions. The search will be updated within one month of submission to publication of the full results. Currently, our team has completed the screening of titles and abstracts, with full text review underway. We anticipate that the full text screening, extraction, and results to be completed by January, March, and June 2026, respectively.

### Source of evidence selection

All articles will be uploaded into Covidence, and duplicates will be removed. Two independent reviewers will review titles and abstracts based on the inclusion criteria (Table 1). Pilot testing of the inclusion and exclusion criteria will be conducted as proposed by JBI [36], with a random selection of 25 articles. Two reviewers will screen based on the eligibility criteria and will subsequently meet to discuss any discrepancies and update the criteria accordingly. The team will only start screening when 80% agreement or greater is achieved. The full text of relevant studies will be retrieved and imported into Covidence. The full text of each study will be reviewed by two independent reviewers based on the same inclusion criteria. Reasons for exclusion of full text articles will be recorded and reported in the review. A third reviewer will be contacted when any conflict arises in the selection process to achieve consensus. The results of the literature search and study inclusion will be summarized in the final scoping review based on the Preferred Reporting Items for Systematic Reviews and Meta-Analyses Extension for Scoping Reviews (PRISMA-ScR) [37].

**Table 1:** Study inclusion and exclusion criteria.

Inclusion criteria		Exclusion criteria
Participants	Adults (18+) with intellectual and/or developmental disabilities receiving cancer-related care Noncancer patients providing support to adults with intellectual and/or developmental disabilities receiving cancer-related care. This may include natural or formal supports (e.g., family members or paid/unpaid caregivers), as well as healthcare professionals (nurses, doctors, etc.)	Studies in which outcomes of individuals with intellectual and/or developmental disabilities cannot be delineated from other groups (e.g., people with disabilities more broadly wherein those with intellectual and/or developmental disabilities experiences or outcomes are not reported respectively)
Concept	Studies exploring outcomes or experiences during the cancer care continuum: awareness/literacy, screening, incidence, stage at diagnosis, treatment, survival, survivorship, psycho-social oncology, supportive care, and end-of-life care	Studies in which cancer outcomes are not reported separately, and or the main focus of the study (e.g., studies exploring other health conditions such as cardiovascular disease, diabetes, etc.) Nonhuman laboratory studies
Type of research	Original research articles (quantitative, qualitative and mixed methods) Articles published from 2006 onwards All settings considered	Grey literature Opinion or commentary articles Editorials Conference abstracts Systematic reviews, meta-analyses, network meta-analyses, narrative reviews, critical reviews, literature reviews, and qualitative reviews Summary report Preprints Case reports or series Archival studies Articles published before 2006

### Data extraction

For each study, data will be extracted on the study setting, participants, study methods, study design and traditions. We will also extract information on cancer outcomes and experiences studied (e.g., awareness/literacy, screening, incidence, stage at diagnosis, treatment, survival, survivorship, psycho-social oncology, supportive care, and end-of-life care) across cancer locations (e.g., breast, colorectal, lung, etc.). We will extract information on whether adults with intellectual and/or developmental disabilities were included in the study, or if a caregiver, family member, or healthcare professional were recruited as a proxy respondent or as primary participants (e.g., study focus was on experiences of family members, caregivers, etc.). We will also extract information on recruitment of adults with intellectual and/or developmental disabilities and the potential for gatekeeping (e.g., participants recruited through service providers, exclusion of individuals without a family advocate, etc.). Information about the person with intellectual and/or developmental disabilities will be extracted, including demographics (e.g., age, sex, race, gender, rurality), underlying health experiences (e.g., other types of conditions such as diabetes or heart disease), and experiences of disability (e.g., level of adaptive supports needed). We will also consider how intellectual and/or developmental disabilities were operationalized (e.g., yes/no, levels of experience/support required in day-to-day life: little support; some support; extensive support, etc.). If a participant without intellectual and/or developmental disabilities is included, we will extract demographic information about that participant and their relationship to the adult with intellectual and/or developmental disabilities, and whether they served as a proxy respondent for the adult with intellectual and/or developmental disabilities or if their experiences as a carer were specifically explored. Specific information will be extracted on how participants were recruited in the study (e.g., qualitative, some types of quantitative studies, etc.) or identified in health databases (e.g., administrative data studies), as well as how people with intellectual and/or developmental disabilities were involved in the study.

We will extract information and organize reporting of engagement of people with intellectual and/or developmental disabilities in each research study based on a community engagement framework developed by International Association for Public Participation (IPA2; Informed, consulted, involved, collaborated, empowered) [35]. First, we will extract details on whether adults with intellectual and/or developmental disabilities and their carers were engaged in the research as advisors or researchers. We will then identify how these advisors were involved in the research. The goals of each public participation category are: 1) *inform*: to provide balanced information to assist with understanding the problem and solutions; 2) *consult*: to get feedback from the persons with lived experience on problem and solutions; 3) *involve*: to work directly with persons with lived experience throughout the project to ensure their perspectives are considered; 4) *collaborate*: to partner with persons with lived experience in each aspect of project decision-making; 5) *empower*: to place decision-making in the hands of persons with lived experience [35]. We will also extract information pertaining to data ownership. If the study focuses on caregivers (paid or unpaid), family members or healthcare professionals, rather than people with intellectual and/or developmental disabilities or involved engagement of these populations as advisors or on the research team, we will also identify how they were involved in the study based on the IPA2 framework. A summary of items for data extraction is available in Table 2. This list will be updated as our team pilots this extraction form for five to ten studies before full extraction. A code book will also be created for resolving ambiguous study details. Data will be extracted by two independent reviewers. Disagreements will be resolved by consultation with a third reviewer.

**Table 2:** Data extraction items.

Item	Description
Study	First Author Year
Title	Title of study
Journal	Journal name
Year	Year study was published
Country	Country of research
Continent	Continent
Funding_sources	Who funded the study
Qual_quant_mixed	Overall study (qual, quant, or mixed methods)
Study_design_tradition	What type of study design (quant) or tradition (qual)
Primary_objective	What is the study objective?
Study_population	Who is included in the study?
IDD_participants	Were there participants with IDD
IDD_participants_text	Who was included
IDD_definition	How was IDD defined?
IDD_operationalized	How was IDD operationalized (e.g., yes/no; level of impairment, etc.)
Others_participants	Were their participants with IDD
Others_participants_text	Who was included
Others_family_friends	Family/friends (unpaid caregivers) included
Others_HCP	Healthcare professionals included
Others_paidcaregiver	Paid caregivers included
N_total	N total
N _IDD	N with IDD
Study_time_frame	Time frame of study
Cancer_site	Cancer sites
Cancer_milestones	Cancer milestones
recruitment_idd_text	How were individuals with IDD recruited?
through_family	Adults with IDD were recruited through family (e.g., materials sent to families directly)
through_services	Adults with IDD were recruited through service organizations
through_other_text	Adults with IDD recruited through another means
Others_recruited_text	How were others without IDD recruited?
consent_idd	Consent procedures described
consent_Idd_text	Pull text- consent process
assent_idd	Assent procedures described
assent_idd_text	Extract text-assent procedures
accessibility	Accessibility in terms of meeting with participants
accessibility_text	Extract relevant text
communication_support	Communication supports discussed in terms of meeting with participants- mentioned
communication_support_text	Pull relevant text
capacity	Details provided regarding assessing capacity (assessed or assumed?)
capacity_tex	Pull relevant text
IDD_gender	Did authors consider gender of individuals with IDD?
IDD_gender_text	What did the authors report/find (e.g., report gender distribution of individuals with IDD, was gender diversity considered, etc.)
IDD_sex	Did authors consider sex of individuals with IDD?
IDD_sex_text	What did the authors report/find (e.g., report sex distribution of individuals with IDD)
IDD_age	Did authors consider age of individuals with IDD?
IDD_age_text	What did the authors report/find (e.g., report age range of those included, etc.)
IDD_comorbidities	Cancer sites (e.g., breast, lung, larger cancer category- e.g., reproductive, GI, B29:C33multiple, etc.)
IDD_comorbidities_text	What did the authors report/find (e.g., number of comorbidities, types, etc.)
IDD_communication	Did authors consider communication level/style of individuals with IDD?
IDD_communication_text	What did the authors report/find (e.g., communication level, etc.)
IDD_race	Did authors consider race of individuals with IDD?
IDD_race_text	What did the authors report/find (e.g., distribution of race, what were the categories, etc.)
IDD_SES	Did authors consider SES of individuals with IDD?
IDD_SES_text	What did the authors report/find (e.g., distribution of SES, what were the categories, etc.)
IDD_region	Did authors consider region of individuals with IDD (e.g., where they live specifically within the given region of the study)?
IDD_region_text	What did the authors report/find (e.g., distribution of region, what were the categories, etc.)
IDD_rural	Did authors consider rurality of individuals with IDD (e.g., how rural is living setting)?
IDD_rural_text	What did the authors report/find (e.g., distribution of rural/urban, what were the categories, etc.)
IDD_living_setting	Did authors consider living setting of individuals with IDD (e.g., in group home, independent, assisted living, etc.)?
IDD_living_setting_text	What did the authors report/find (e.g., distribution of independent, group living, etc.)
IDD_adl	Did the authors consider adaptive daily living skills of individuals with IDD (more support, less support, etc.)
IDD_adl_text	What did the authors report/find (e.g., distribution of more/less supprot for ADLs, etc.
Key_findings	Main key finding from the study
Strengths	Main strengths of the study
Limitations	Main limitations
admin_data	Was admin data used
medical_sources_only	For admin data- were the sources only medical (e.g., no social, disability, etc. data sources)
Data_sources	For admin data- List data sources included
Diagnostic_codes	For admin data- List diagnostic codes included in algorithm
visits_ algorithm	For admin data- Note visits, etc. for the algorithm for identifying IDD status
Quant_outcomes_measurement	For admin and non-admin data- If quant- what were the main outcomes that were examined?
main_analysis_type	For admin and non-admin data- Main statistical analyses that were conducted
Statistical_analysis_main_finding	For admin and non-admin data- Main finding from the primary statistical analysis
Confounders	For admin and non-admin data- Confounders that were adjusted for
Effect_modification	For admin and non-admin data- Effect modification that was considered (e.g., which variables were analyses stratified by)
Qual_experiences	Were qual experiences explored
Qual_analysis	What was the qual analysis type (e.g., thematic analysis, etc.)
Qual_main finding	What was the primary finding from the qualitative study
enagement_idd	Adults with IDD were in the study as participants, advisors, or co-researchers
engagement_carer	Carers of adults with IDD were in the study as participants, advisors, or co-researchers
Inform	Team provided people with IDD with balance and objective information to assist them in understanding the problems, alternatives and/or solutions.
Inform_text	If yes- provided description
Consult	Team provided opportunity to obtain feedbackfrom individuals with IDD on analysis, alternatives and/or decisions.
Consult_text	If yes- provided description
Involve	Team worked directly with people with IDD throughout the process to ensure that their concerns and aspirations are consistently understood and considered.
Involve_text	If yes- provided description
Collaborate	Team partnered with individuals with IDD in each aspect of the decision, including the development of alternatives and the identification of preferred solution.
Collaborate_text	If yes- provided description
Empower	Team placed final decision-making in the hands of the persons with IDD.
Empower_text	If yes- provided description
Inform_family	Team provided family members with balance and objective information to assist them in understanding the problems, alternatives and/or solutions.
Inform_family_text	If yes- provided description
Consult_family	Team provided opportunity to obtain feedback from family members on analysis, alternatives and/or decisions.
Consult_family_text	If yes- provided description
Involve_family	Team worked directly with family members throughout the process to ensure that their concerns and aspirations are consistently understood and considered.
Involve_family_text	If yes- provided description
Collaborate_family	Team partnered with family members in each aspect of the decision, including the development of alternatives and the identification of preferred solution.
Collaborate_family_text	If yes- provided description
Empower_family	Team placed final decision-making in the hands of the family members.
Empower_family_text	If yes- provided description

### Data analysis and presentation

Descriptive statistics will be used to summarize study characteristics (type of study, cancer site, type of study design/tradition). We will display how adults with intellectual and/or developmental disabilities were identified in administrative data sources, including diagnoses used to identify intellectual and/or developmental disabilities, across population-based studies. We will present different definitions of intellectual and/or developmental disabilities across studies and outline gaps in the research conducted. Our team will also summarize information provided about adults with intellectual and/or developmental disabilities and their carers, including the extent to which intersectionality is captured or considered (e.g., the intersection of disability and race/ethnicity). Intersectionality will be considered within the context of the social determinants of health noted by the World Health Organization [41]. We will synthesize if and how adults with intellectual and/or developmental disabilities were recruited into studies that involved primary data collection, and whether those involved in their care were recruited as well. Descriptions of consent will be analyzed to determine the extent to which strategies proposed by McDonald et al. (2024) are followed for responsible inclusion [42]. We will display how adults with intellectual and/or developmental disabilities and their family members, caregivers (paid or unpaid), or healthcare professionals were engaged in each study as advisors or co-researchers based on the IPA2 framework [35], namely if participants were informed, consulted, involved, collaborated with, or empowered. Results will be summarized by mapping findings on cancer care milestones. For example, we will consider the representation, involvement and engagement of adults with intellectual and/or developmental disability and their carers in research focused on cancer awareness, prevention, screening, staging, treatment, survival, and end-of-life care. We will also explore changes over time to consider how representation, involvement and engagement of this patient population in cancer-related research have changed over time. Our team will work with our advisors to develop easy read summaries that are accessible when presenting findings to adults with intellectual and/or developmental disabilities and those involved in their care.

### Data

Relevant data from this study will be made available upon request following completion and publication.

## Supporting information

S1 Table Intellectual and/or developmental disability diagnostic codes from International classification of disease 9 and 10. (DOCX)

S2 Table EMBASE search. (DOCX)

S3 Table Medline search. (DOCX)

S4 Table PyschINFO search. (DOCX)

S5 Table CINAHL search. (DOCX)
