# Supplementary material for: How are adults with intellectual and/or developmental disabilities represented, included and engaged in cancer research: A scoping review protocol
Source: PLoS One. 2026 Apr 15;21(4):e0346010. doi: 10.1371/journal.pone.0346010 (PMC13082627; doi:10.1371/journal.pone.0346010)
Supplement: S1 Table — (DOCX) [file pone.0346010.s001.docx]

# Table S1. Intellectual and/or developmental disability diagnostic codes from International Classification of Disease 9 and 10.

| **Diagnostic Code (ICD-9)** | **Description** |
| --- | --- |
| 299-2999 | Pervasive development disorders (e.g., autism) |
| 317-3179 | Mental retardation |
| 318-3189 | Mental retardation |
| 319-3199 | Mental retardation |
| 7580-7583 | Chromosomal anomalies for which a developmental disability is typically present |
| 7585 | Other conditions due to autosomal anomalies |
| 7588 | Other conditions due to chromosome anomalies |
| 7589 | Conditions due to anomaly of unspecified chromosome |
| 7595 | Tuberous sclerosis |
| 75981 | Other and unspecified congenital anomalies: Prader Willi |
| 75982 | Marfan Syndrome |
| 75983 | Other and unspecified congenital anomalies: fragile x |
| 75989 | Other and unspecified congenital anomalies: other  (e.g. menkes disease, Laurence-Moon-Biedl,rubinstein-taybi syndrome etc.) |
| 76070 | NOXIOUS SUBST NOS AFF NB |
| 76071 | NOXIOUS SUBST NOS AFF NB |
| 76072 | MATERNAL ALCOHOL AFF NB |
| 76072 | MATERNAL NARCOTIC AFF NB |
| 76073 | MATERNAL HALLUCIN AFF NB |
| 76074 | MATERNAL ANTI-INF AFF NB |
| 76075 | COCAIN AFF FET VIA PLAC/BR MILK |
| 76077 | Fetal hydantoin syndrome |
| 76079 | NOXIOUS SUBST NEC AFF NB |
| **Diagnostic Code (ICD-10)** | **Description** |
| F70/F700 | Mild mental retardation with the statement of no, or minimal, impairment of behaviour |
| F701 | Mild mental retardation, significant impairment of behaviour requiring attention or treatment |
| F708 | Mild mental retardation, other impairments of behaviour |
| F709 | Mild mental retardation without mention of impairment of behaviour |
| F71/F710 | Moderate mental retardation with the statement of no, or minimal, impairment of behaviour |
| F711 | Moderate mental retardation, significant impairment of behaviour requiring attention or treatment |
| F718 | Moderate mental retardation, other impairments of behaviour |
| F719 | Moderate mental retardation without mention of impairment of behaviour |
| F72/F720 | Severe mental retardation with the statement of no, or minimal, impairment of behaviour |
| F721 | Severe mental retardation, significant impairment of behaviour requiring attention or treatment |
| F728 | Severe mental retardation, other impairments of behaviour |
| F729 | Severe mental retardation without mention of impairment of behaviour |
| F73/F730 | Profound mental retardation with the statement of no, or minimal, impairment of behaviour |
| F731 | Profound mental retardation, significant impairment of behaviour requiring attention or treatment |
| F738 | Profound mental retardation, other impairments of behaviour |
| F739 | Profound mental retardation without mention of impairment of behaviour |
| F78/F780 | Other mental retardation with the statement of no, or minimal, impairment of behaviour |
| F781 | Other mental retardation, significant impairment of behaviour requiring attention or treatment |
| F788 | Other mental retardation, other impairments of behaviour |
| F789 | Other mental retardation without mention of impairment of behaviour |
| F79/F790 | Unspecified mental retardation with the statement of no, or minimal, impairment of behaviour |
| F791 | Unspecified mental retardation, significant impairment of behaviour requiring attention or treatment |
| F798 | Unspecified mental retardation, other impairments of behaviour |
| F799 | Unspecified mental retardation without mention of impairment of behaviour |
| F840 | Childhood autism |
| F841 | Atypical autism |
| F842 | Rett’s Syndrome |
| F844 | Overactive disorder associated with mental retardation and stereotyped movements |
| F845 | Asperger’s syndrome |
| F848 | Other pervasive developmental disorders |
| F849 | Pervasive developmental disorder, unspecified |
| Q851 | Tuberous sclerosis |
| Q860 | Fetal Alcohol Syndrome |
| Q861 | Fetal hydantoin syndrome |
| Q871 | Aarskog, Prader willi, DeLange, Seckel etc. |
| Q878 | Other |
| Q900-Q939 **except** Q926 | (i.e., all Down Syndrome Types, cri du chat, etc **except** Extra marker chromosomes) |
| Q971 | Female with more than three X chromosomes |
| Q992 | Fragile X |
| Q998 | Other specified chromosome abnormalities |
