# Supplementary material for: How are adults with intellectual and/or developmental disabilities represented, included and engaged in cancer research: A scoping review protocol
Source: PLoS One. 2026 Apr 15;21(4):e0346010. doi: 10.1371/journal.pone.0346010 (PMC13082627; doi:10.1371/journal.pone.0346010)
Supplement: S2 Table — (DOCX) [file pone.0346010.s002.docx]

# Table S2. EMBASE search.

**Database: Embase Classic+Embase <1947 to 2025 August 08>**
**Search Strategy:**
**1**  exp neoplasm/ (7087169)
**2**  cancer.mp. (5200321)
**3**  carcinoma.mp. (1672198)
**4**  tumo?r*.mp. (4709814)
**5**  1 or 2 or 3 or 4 (8629150)
**6**  developmental delay/ (23660)
**7**  intellectual impairment/ (49824)
**8**  mental deficiency/ (81032)
**9**  developmental disorder/ (43859)
**10**  developmental disabilit*.mp. (11539)
**11**  intellectual disabilit*.mp. (44040)
**12**  IDD.mp. (5461)
**13**  (intellectual adj3 developmental disabilit*).mp. [mp=title, abstract, heading word, drug trade name, original title, device manufacturer, drug manufacturer, device trade name, keyword heading word, floating subheading word, candidate term word] (3096)
**14**  or/6-13 (198579)
**15**  5 and 14 (17450)
**16**  limit 15 to (adult or aged ) (4992)
**17**  limit 16 to yr="2006 - 2025" (4169)
