# Supplementary material for: How are adults with intellectual and/or developmental disabilities represented, included and engaged in cancer research: A scoping review protocol
Source: PLoS One. 2026 Apr 15;21(4):e0346010. doi: 10.1371/journal.pone.0346010 (PMC13082627; doi:10.1371/journal.pone.0346010)
Supplement: S3 Table — (DOCX) [file pone.0346010.s003.docx]

# Table S3. Medline search.

**Database: Ovid MEDLINE(R) ALL <1946 to August 08, 2025>**
**Search Strategy:**
**1**  exp Neoplasms/ (4141198)
**2**  cancer.mp. (2510022)
**3**  carcinoma.mp. (1054420)
**4**  tumo?r*.mp. (2774693)
**5**  1 or 2 or 3 or 4 (5596269)
**6**  Developmental Disabilities/ (23728)
**7**  exp Intellectual Disability/ (110513)
**8**  developmental disabilit*.mp. (28626)
**9**  intellectual disabilit*.mp. (85256)
**10**  IDD.mp. (4048)
**11**  (intellectual adj3 developmental disabilit*).mp. [mp=title, book title, abstract, original title, name of substance word, subject heading word, floating sub-heading word, keyword heading word, organism supplementary concept word, protocol supplementary concept word, rare disease supplementary concept word, unique identifier, synonyms, population supplementary concept word, anatomy supplementary concept word] (2268)
**12**  6 or 7 or 8 or 9 or 10 or 11 (154728)
**13**  5 and 12 (6893)
**14**  limit 13 to "all adult (19 plus years)" (1810)
**15**  limit 14 to yr="2006 - 2025" (933)
