# Supplementary material for: How are adults with intellectual and/or developmental disabilities represented, included and engaged in cancer research: A scoping review protocol
Source: PLoS One. 2026 Apr 15;21(4):e0346010. doi: 10.1371/journal.pone.0346010 (PMC13082627; doi:10.1371/journal.pone.0346010)
Supplement: S4 Table — (DOCX) [file pone.0346010.s004.docx]

# Table S4. PyschINFO search.

**Database: APA PsycInfo <1806 to August 2025 Week 1>**
**Search Strategy:**
**1**  exp neoplasms/ (69215)
**2**  cancer.mp. (80709)
**3**  carcinoma.mp. (2798)
**4**  tumo?r*.mp. (25067)
**5**  1 or 2 or 3 or 4 (104790)
**6**  exp intellectual development disorder/ (52225)
**7**  developmental disabilities/ (14733)
**8**  developmental disabilit*.mp. (23643)
**9**  intellectual disabilit*.mp. (35089)
**10**  IDD.mp. (1532)
**11**  (intellectual adj3 developmental disabilit*).mp. [mp=title, abstract, heading word, table of contents, key concepts, original title, tests & measures, mesh word] (3133)
**12**  or/6-11 (80783)
**13**  5 and 12 (487)
**14**  limit 13 to "300 adulthood (age 18 yrs and older)" (198)
**15**  limit 14 to yr="2006 - 2025" (158)
