# Supplementary material for: How are adults with intellectual and/or developmental disabilities represented, included and engaged in cancer research: A scoping review protocol
Source: PLoS One. 2026 Apr 15;21(4):e0346010. doi: 10.1371/journal.pone.0346010 (PMC13082627; doi:10.1371/journal.pone.0346010)
Supplement: S5 Table — (DOCX) [file pone.0346010.s005.docx]

# Table S5. CINAHL search.

| **rch ID#** | **Search Terms** | **Results** |
| --- | --- | --- |
| S15 | S10 AND S14 | 322 |
| S14 | S1 OR S2 OR S12 OR S13 | 906,272 |
| S13 | "tumo?r*" | 109,358 |
| S12 | "carcinoma" | 139,640 |
| S11 | S3 AND S10 | 378 |
| S10 | S4 OR S5 OR S6 OR S7 OR S8 OR S9 | 57,769 |
| S9 | intellectual n3 developmental disabilit* | 2,588 |
| S8 | IDD | 20,123 |
| S7 | intellectual disabilit* | 34,347 |
| S6 | developmental disabilit* | 15,407 |
| S5 | (MH "Developmental Disabilities") | 12,268 |
| S4 | (MH "Intellectual Disability+") | 38,503 |
| S3 | S1 OR S2 | 875,086 |
| S2 | cancer | 591,867 |
| S1 | (MH "Neoplasms+") | 678,284 |
